# Effects of Sambiloto (*Andrographis paniculata*) on GLP-1 and DPP-4 Concentrations between Normal and Prediabetic Subjects: A Crossover Study

**DOI:** 10.1155/2022/1535703

**Published:** 2022-01-15

**Authors:** Tri Juli Edi Tarigan, Erni Hernawati Purwaningsih, Yusra, Murdani Abdullah, Nafrialdi, Joedo Prihartono, Made Ratna Saraswati, Imam Subekti

**Affiliations:** ^1^Division of Endocrinology and Metabolism, Department of Internal Medicine, Dr. Ciptomangunkusumo National General Hospital, Faculty of Medicine, Universitas Indonesia, Jakarta, Indonesia; ^2^Department of Medical Pharmacy, Faculty of Medicine, Universitas Indonesia, Jakarta, Indonesia; ^3^Department of Clinical Pathology, Dr. Ciptomangunkusumo National General Hospital, Faculty of Medicine, Universitas Indonesia, Jakarta, Indonesia; ^4^Division of Gastroenterology, Department of Internal Medicine, Dr. Ciptomangunkusumo National General Hospital, Faculty of Medicine, Universitas Indonesia, Jakarta, Indonesia; ^5^Department of Pharmacology, Faculty of Medicine, Universitas Indonesia, Jakarta, Indonesia; ^6^Department of Community Medicine, Faculty of Medicine, Universitas Indonesia, Jakarta, Indonesia; ^7^Division of Endocrinology and Metabolism, Department of Internal Medicine, Sanglah Hospital, Faculty of Medicine, Universitas Udayana, Bali, Indonesia

## Abstract

**Background:**

The extract of *Andrographis paniculata* (Burm. F.) Wall. Ex. Nees. (sambiloto) (穿心蓮 chuān xīn lián) has been reported to have an antidiabetic effect on mice models and has been used traditionally in the community. The exact mechanism of sambiloto extract in decreasing plasma glucose is unclear, so we investigated the role of sambiloto extract in the incretin pathway in healthy and prediabetic subjects.

**Methods:**

This study was a randomized, placebo-controlled, crossover, double-blind trial. It included 38 people who were healthy and 35 people who had prediabetes. All subjects were randomly assigned to receive either the intervention sambiloto extract or a placebo. All subjects were randomly assigned to receive the first intervention for 14 days. There was a washout period between subsequent interventions. The primary outcome was glucagon-like peptide 1 (GLP-1) concentration, and secondary outcomes were fasting insulin, 2-hour postprandial insulin, homeostasis model assessment of insulin resistance (HOMA-IR), fasting blood glucose, 2-hour postprandial blood glucose, dipeptidyl peptidase-4 (DPP-4), and glycated albumin before and after the intervention.

**Result:**

After the intervention, GLP-1 concentration significantly increased in prediabetes by 19.6% compared to the placebo (*p*=0.043). There were no significant differences in the changes of fasting insulin, 2-hour postprandial insulin, HOMA-IR, fasting blood glucose, 2-hour postprandial blood glucose, DPP-4, and glycated albumin levels after the intervention. Sambiloto extract did not inhibit the DPP-4 enzyme in healthy and prediabetic subjects.

**Conclusion:**

Sambiloto extract increased GLP-1 concentration without inhibiting the DPP-4 enzyme in prediabetic subjects. This trial is registered with ClinicalTrials.gov (ID: NCT03455049), registered on 6 March 2018—retrospectively registered (https://clinicaltrials.gov/ct2/show/NCT03455049).

## 1. Introduction

Type 2 diabetes mellitus (T2DM) affects approximately 8.5 percent of the global population or 415 million people. It is expected to increase to 642 million by 2040 [1]. Many groups of antidiabetic drugs are available, but many of them have unfavorable side effects, such as hypoglycemia. Therefore, the quest for an ideal treatment for T2DM continues. The most current and extensively studied treatment of T2DM is incretin-based therapy.

Incretins are hormones released from the small intestine into the bloodstream in response to food intake, especially carbohydrates. The main incretin hormones produced in the intestine are glucagon-like peptide 1 (GLP-1) and glucose-dependent insulinotropic peptide (GIP), which are produced mainly in ileal L cells and jejunum *K* cells, respectively. Incretin hormones mediate the insulinotropic response of intestinal nutrients [2].

From the perspective of incretin activity, there is robust evidence regarding the decrease of the incretin effect in T2DM, but the exact mechanism remains unknown. There are several strategies to increase the incretin effect, such as administering exogenous subcutaneous GLP-1, oral administration of dipeptidyl peptidase-4 (DPP-4) enzyme inhibitors, and oral administration of small-molecule GLP-1 receptor ligands. A GLP-1 receptor ligand is a small molecule that can bind to a part of the GLP-1 receptor (GLP-1R); therefore, the biological effect due to GLP-1 and GLP-1 receptor binding becomes more optimal, thus increasing insulin secretion. Several phytochemicals were found to act as a GLP-1 receptor ligand. However, the number of evidence is limited [3].

Phytotherapy has been developed for T2DM treatment with a variety of pharmacodynamics and pharmacokinetic properties. In addition, it has a minimal hypoglycemic effect. However, only a few are accepted scientifically and have been evaluated for clinical effectiveness [4]. The phytochemical andrographolide and flavonoid (polyherbal) mixture is more popular than the other mixtures for its hypoglycemic effect [5,6]. Flavonoids are small molecules that can act as ligands at the GLP-1 receptor and subsequently stimulate insulin production and secretion [3]. Natural and synthetic flavonoids can modulate GLP-1R in pancreatic beta cells; therefore, more calcium ions can be transported intracellularly and further release insulin [7]. In addition to the flavonoid, andrographolide is the active ingredient of the diterpene lactone component, which has a hypoglycemic and antioxidant effect [8]. Andrographolide works through the GLP-1 pathway as a voltage amplifier dependent on the potassium channel (*K* + *v*), both on the triggering and amplifying pathway [9]. Andrographolide also has a hydroxyl group and is suspected as a ligand of the GLP-1 receptor and modulates insulin production through increasing intracellular calcium [10].

*Andrographis paniculata* (Burm. F.) Wall. Ex. Nees. (sambiloto) (穿心蓮 chuān xīn lián), is one of the plants that contain andrographolide and flavonoids, which is traditionally used as an antidiabetes drug. Besides andrographolide, which is the major one in terms of bioactive properties and abundance, the other components that could be found in this plant are as follows [11]:

1. Andrographolide analogues: 14-deoxy-11,12-didehydroandrographolide, neoandrographolide, 14-deoxyandrographolide
2. Andrograpanin
3. 14-Deoxy-14,15-dehydroandrographolide
4. Isoandrographolide
5. 3,19-Isopropylideneandrographolide
6. 14-Acetylandrographolide
7. Arabinogalactan proteins
8. Flavonoids: 7-*O*-methylwogonin, apigenin, onysilin, and 3,4-dicaffeoylquinic acid

Numerous studies have proved the hypoglycemic effects in andrographolide and flavonoids in animals and humans; however, there is no available human study with the RCT and crossover method to find the mechanism of action of sambiloto in lowering blood glucose. In addition, studies regarding the role of GLP-1 receptor ligand (incretin enhancers) properties in andrographolide and flavonoid are minimal. Therefore, it is necessary to find small-molecular GLP-1 receptor ligand orally that have minimal side effects as a new antidiabetic drug.

## 2. Methods

### 2.1. Study Design

This study was a randomized, double-blind, crossover design, placebo-controlled trial conducted in Jakarta, Indonesia, at Dr. Cipto Mangunkusumo National Referral Hospital, Makara Health Centre Universitas Indonesia, and a taxi company. The intervention was assigned using permuted block randomization with a two-block size combination of sambiloto and placebo. The study was conducted following the Helsinki Declaration and the International Conference on Harmonization's Guidelines for Good Clinical Practice. The Medical Research Ethics Committee of Universitas Indonesia's Faculty of Medicine approved the study protocol. This trial is also registered with ClinicalTrials.gov (NCT03455049).

### 2.2. Subjects

All subjects received written informed consent and signed it before any study procedures occurred. A total of 184 people were screened. Seventy-three subjects met the inclusion criteria. All subjects were enrolled and divided into two groups: healthy and those who had prediabetes. All subjects were then randomly assigned to either a sambiloto or a placebo intervention.

Inclusion criteria were as follows:

1. 18- to 60-year-old male and female
2. Classified as healthy or prediabetic subjects by oral glucose tolerance test (OGTT)
3. Normal liver and kidney function: a minimum sample size of 35 prediabetic and healthy subjects in each group provided a power of 80% with the assumption of a significance level of 0.05

Exclusion criteria were as follows:

1. Pregnant
2. Breastfeeding
3. Severe concomitant diseases and uncontrolled chronic diseases
4. Diagnosed as malignancy disease
5. Consumption of drugs that might affect blood glucose levels, such as steroids and herbal supplements (bitter melon, red ginseng, cinnamon, *brotowali*, and bay leaves)

### 2.3. Measurement

The subjects were examined three times during 35 days of the intervention period: screening, visit 1, visit 2 (14 days after the first intervention), and visit 3 (14 days after the second intervention, the end of the study). This study assessed GLP-1, fasting insulin, 2-hour postprandial insulin, homeostasis model assessment of insulin resistance (HOMA-IR), fasting blood glucose, 2-hour postprandial blood glucose, glycated albumin, and DPP-4 before and after the intervention.

### 2.4. Treatments

Borobudur Herbal Company provided 550 mg of sambiloto extract capsules for this study. The capsules were sent to the lab for further analysis to determine the concentration of andrographolide and flavonoid. The spectrophotometry and HPLC methods were used for the analysis. It was discovered that each capsule contains 1.2% andrographolide and 0.8% flavonoid. The placebo contains 98% lactose and 2% magnesium.

### 2.5. Statistical Analyses

Descriptive data were expressed as mean (standard deviation) when the data are normally distributed and median (minimal-maximal) when the data are not normally distributed. The difference in outcome parameters between interventions was assessed using a paired *T*-test or Wilcoxon test. Significance was considered at *p* < 0.05. All statistical analyses were performed using SPSS version 20.0 (IBM SPSS Inc., Chicago, USA). To analyze the possible mechanism of sambiloto extract in glucose metabolism, we used path analysis with Stata.

## 3. Results

### 3.1. Subject Characteristics

**Figure 1:**
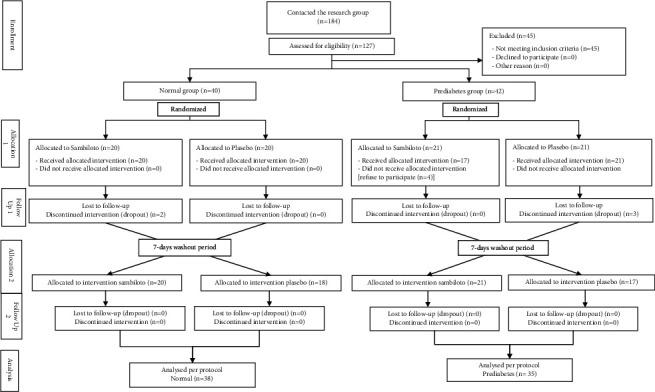
Consort flow diagram.

Among the 73 participants who completed the study protocol, thirty-eight subjects were in the healthy group, and 35 subjects were in the prediabetes group (Figure 1). The characteristics of the subjects are summarized in Table 1. Table 1 shows the mean age of the prediabetes group was 47.03 (±8.52) years old, ten years older than the healthy subject group which was 37.55 (±9.29) years old, and 52 (71.23%) of the subjects were male. The obesity rate was higher in the prediabetes group (85.70%) than in the healthy group (60.50%). Prediabetic subjects have a higher waist circumference when compared to the healthy group. Smoking habits were found more in prediabetic subjects (65.80%) when compared to healthy subjects (13.20%).

### 3.2. Changes of Parameter Values in Healthy Subjects

After the sambiloto extract or placebo intervention, the levels of GLP-1, fasting insulin, fasting blood glucose, DPP-4 enzyme, and HOMA-IR were increased, although these changes were not statistically significant. However, the levels of two-hour postprandial insulin, two-hour postprandial blood glucose, and glycated albumin were decreased, although these changes were not statistically significant. The summary is expressed in Table 2.

### 3.3. Changes of Parameter Values in Prediabetic Subjects

There was a statistically significant increase in GLP-1 levels in prediabetic subjects after intervention (*p*=0.043) compared with placebo. Changes also occurred in other parameters but were not found to be statistically significant. Changes in parameters after intervention can be seen in Table 3.

### 3.4. Safety

There were five adverse events (2 participants from the healthy group and 3 participants from the prediabetes group). Complaints were hand tremors, red spots on faces, itchiness, lethargy, weakness, diarrhea, and palpitation after consuming the capsules. All the subjects were withdrawn from the study and considered dropout. All events were recorded and submitted in a written report to the ethical committee. There were no serious adverse events that occurred during clinical trials.

## 4. Discussion

Most of the subjects in this study were male, similar to a previous study completed by Soewondo et al. [12] which identified male gender as a predictive factor for prediabetes in Indonesia (OR 0.8). This finding was due to the fact that most of the subjects screened were taxi drivers (57.60%). Most of the prediabetic subjects were in obese and overweight conditions. The study by Sirait et al. [13] found that the risk of T2DM in mild obesity was two times greater, moderate obesity was five times greater, and severe obesity was ten times greater than in nonobese individuals. Waist circumference was also shown to be more significant in the prediabetic subjects compared to healthy subjects. From the description of the characteristics, it can be concluded that cardiovascular risk factors were more commonly found in prediabetic subjects. According to the study by Michaliszyn et al. [14], beta-cell glucose sensitivity decreased 30% in prediabetes and 65% in T2DM, similar to the incretin effect that decreases 32% in prediabetes and 38% in T2DM.

### 4.1. Effects of Sambiloto (*Andrographis paniculata*) Extract on GLP-1 and DPP-4 Enzyme Levels

This study showed that administration of sambiloto extract in healthy subjects increased GLP-1 levels, but the increasing GLP-1 levels also occurred in the placebo group. These findings indicate that the sambiloto effect does not cause an increased GLP-1 level but other confounding factors such as the physiological response of postprandial glucose testing with 75 grams of oral glucose solution.

The administration of sambiloto extract in the prediabetic subjects increased GLP-1 levels by 19.6% and was statistically significant compared to placebo (*p*=0.043). These results were similar to those of the study by Purnomo et al. in diabetic rats given *Urena lobata*, a type of herb that contains flavonoids that can increase GLP-1 levels [15].

This study found an increase in DPP-4 enzyme levels in both healthy and prediabetic subjects. The purpose of testing DPP-4 enzyme levels in this study was to show that the mechanism of action of sambiloto extract was through the GLP-1 pathway and not through the inhibition of the DPP-4 enzyme. If the sambiloto extract worked through the DPP-4 enzyme inhibition, it was expected that there would be a decrease in the enzyme [16]. In this study, we found an increase in GLP-1 and DPP-4 enzymes. As a result, it is possible to conclude that the sambiloto extract works via the GLP-1 pathway rather than by inhibiting the DPP-4 enzyme. This result was similar to the study conducted by Riyanti that the *Andrographis paniculata* extract only inhibits the DPP-4 enzyme by 37%, while other plants such as *Trigonella foenum-graecum L* can inhibit the DPP-4 enzyme by 71% [17].

### 4.2. Effects of Sambiloto (*Andrographis paniculata*) Extract on Fasting Insulin Levels and HOMA-IR

The fasting insulin levels increased after the administration of sambiloto extract and placebo in the healthy subjects. This result was in line with the increasing HOMA-IR in the healthy subjects, reflecting insulin resistance status. In healthy subjects, insulin resistance status can change rapidly because of many factors such as physical activity, food intake, psychological condition, the presence of other metabolic stresses, and hormonal changes associated with glucose and insulin homeostasis.

In the prediabetic subjects, fasting insulin levels had no change after administering sambiloto extract. However, there was a decrease in HOMA-IR. In contrast, the administration of a placebo increased the fasting insulin level and was followed by a decrease of HOMA-IR. These findings suggest that the decreasing level of fasting insulin could follow the improvement of insulin resistance. This finding is similar to a study in diabetic rats that showed andrographolide can ameliorate HOMA-IR [18].

### 4.3. Effect of Sambiloto *(Andrographis paniculata)* Extract on 2-Hour Postprandial Insulin Level

Two-hour postprandial insulin levels decreased, followed by a decrease of blood glucose levels after intervention with sambiloto and placebo in healthy subjects. A decrease in insulin levels in this study was not necessarily followed by an increase in blood glucose levels due to many confounding factors such as counterregulatory hormones, peripheral glucose uptake, and insulin levels in the blood [19].

There was a decrease of 2-hour postprandial insulin levels followed by an increase of 2-hour postprandial blood glucose levels after intervention with sambiloto and placebo in prediabetic subjects, therefore suggesting a decrease in insulin would further increase the level of blood glucose. A decrease in postprandial insulin levels was not appropriate as there was an increase of GLP-1 levels in prediabetic subjects. It was difficult to answer this discrepancy due to the unavailable serial examinations of the blood glucose, GLP-1, and insulin levels. A serial examination would demonstrate the dynamics of each parameter; therefore, we can observe the exact interactions between the three parameters. Some factors can influence the postprandial insulin level, such as a standardized daily calorie intake, physical activity before the examination, and psychological factors of the subject. Those factors were not standardized in this study [20–22].

### 4.4. Effects of Sambiloto *(Andrographis paniculata)* Extract on Glycemic Control

In both groups, healthy and prediabetes, there was a decrease in glycated albumin for 14 days of treatment. However, the changes were not statistically significant when compared to placebo. This finding suggested that during treatment, the average blood glucose level was decreasing.

In healthy subjects, there was an increase in fasting blood glucose in both interventions. This finding suggested that in healthy subjects, the homeostasis of blood glucose is still adequate to compensate for any changes of hormonal conditions. In prediabetic subjects, fasting blood glucose also decreased in both interventions and was consistent with a decrease in glycated albumin. This finding was similar to a study in prediabetic rats given *Psidium guajava*, the herb containing flavonoid [23]. Another study explained that andrographolide could inhibit gluconeogenesis, which was subsequently followed by a decrease in fasting blood glucose [24].

The decrement of 2-hour postprandial blood glucose after the intervention, either sambiloto extract or placebo, was found in healthy subjects. In contrast to prediabetes, there was a decrease of 2-hour postprandial insulin followed by an increase of 2-hour postprandial blood glucose. This finding was unexpected because there was an increment of GLP-1 after receiving sambiloto intervention. This phenomenon could be explained by GLP-1 resistance in prediabetes, in which GLP-1 fails to stimulate insulin production, increasing 2-hour postprandial blood glucose levels. Many studies reported GLP-1 resistance in prediabetes and T2DM. Therefore, one method of diabetes treatment today is to increase the incretin effect [25, 26].

## 5. Conclusion

The extract of sambiloto increased GLP-1 concentration without inhibiting the DPP-4 enzyme in prediabetic subjects. The increase of GLP-1 concentration happened through another mechanism without inhibiting the DPP-4 enzyme. Further research needs to be done to see whether the extract of sambiloto has any in-vitro activity on the DPP4 enzyme.

## Figures and Tables

**Table 1:** Subject characteristics.

	Normal (*n* = 38)	Prediabetes (*n* = 35)
Age (years), mean (SD)	37.6 (9.3)	47.0 (8.5)
Gender, *n* (%)		
Male	22 (57.9)	30 (85.7)
Female	16 (42.1)	5 (14.3)
Smoking status, *n* (%)		
Yes	5 (13.2)	23 (65.8)
No	33 (86.8)	12 (34.2)
Family history of T2DM, *n* (%)		
Yes	13 (34.2)	7 (20)
No	25 (65.8)	28 (80)
Family history of hypertension, *n* (%)		
Yes	8 (21.1)	15 (42.9)
No	30 (78.9)	20 (57.2)
Family history of dyslipidemia, *n* (%)		
Yes	5 (13.2)	3 (8.6)
No	33 (86.8)	32 (91.4)
BMI (kg/m^2^), *n* (%)		
Underweight (<18.5)	3 (7.9)	(-)
Normal (18.5–22.9)	7 (18.4)	1 (2.9)
Overweight (23–24.9)	5 (13.2)	4 (11.4)
Obesity grade I and II (≥25)	23 (60.5)	30 (85.7)
Waist circumference (cm), mean (SD)		
Male	86.334 (17.9)	92.07 (12.9)
Female	80.955 (15.6)	91.50 (10.6)
Laboratory test		
ALT, U/L median (min.–max.)	19.50 (7–73)	25 (9–131)
Creatinin (mg/dL), median (min.–max.)	1.00 (0–1.0)	1.00 (0.50–1.8)
eGFR (mL/1.73 m^2^/minute), mean (SD)	112.32 (20.0)	84.71 (12.9)

SD, standard deviation; min-max, minimum-maximum; T2DM, type 2 diabetes mellitus; BMI, body mass index; ALT, alanine aminotransferase; eGFR, estimated glomerular filtration rate.

**Table 2:** Comparison of parameters before and after sambiloto or placebo interventions in the normal subjects (*n* = 38).

Parameter	Presambiloto	Postsambiloto	Δ sambiloto	Preplacebo	Postplacebo	Δ placebo	*p* value Δ
GLP-1 (pmol/L), median (min.–max.)	1.9 (0.8–15.4)	2.9 (0.7–12.4)	0.4 (−11.9–10.5)	1.9 (0.8–15.4)	2.8 (0.7–9.9)	0.5 (−12.7–8.2)	0.911^a^
Fasting insulin (mU/L), mean (SD)	16.5 (12.1)	18.1 (9.2)	1.6 (6.7)	16.5 (12.1)	17.6 (11.0)	1.1 (7.5)	0.605^a^
2-hour postprandial insulin (mU/L), mean (SD)	65.2 (44.1)	65.1 (43.6)	−0.1 (52.0)	65.2 (44.1)	62.1 (35.8)	−3.1 (39.6)	0.613^a^
HOMA-IR, mean (SD)	3.3 (2.7)	3.7 (2.2)	0.4 (1.6)	3.3 (2.7)	3.5 (2.4)	0.2 (1.7)	0.204^a^
FBG (mg/dL), mean (SD)	78.79 (8.91)	80.55 (9.17)	1.76 (8.18)	78.79 (8.91)	80.08 (8.97)	1.29 (8.52)	0.691^a^
2-hour postprandial blood glucose (mg/dL), mean (SD)	93.84 (20.94)	93.71 (26.38)	−0.13 (29.51)	93.84 (20.94)	88.39 (23.95)	−5.45 (25.44)	0.253^a^
DPP-4 (ng/mL), mean (SD)	421.51 (115.35)	431.04 (110.31)	9.53 (65.80)	421.51 (115.35)	433.11 (123.29)	11.59 (61.78)	0.838^a^
GA (%), mean (SD)	12.54 (1.12)	11.63 (1.29)	−0.91 (1.17)	12.54 (1.12)	11.63 (1.44)	−0.91 (1.00)	0.973^a^

Notes: comparative analysis using^a^ paired t-test. GA, glycated albumin.

**Table 3:** Comparison of parameters before and after sambiloto or placebo interventions in prediabetic subjects (*n* = 35)

Parameter	Presambiloto	Postsambiloto	Δ sambiloto	Preplacebo	Postplacebo	Δ placebo	*p* value Δ
GLP-1 (mol/L), median (min.–max.)	2.7 (0.2–40.1)	3.2 (1.0–35.5)	0.3 (-4.6–5.9)	2.7 (0.2–40.1)	2.96 (0.6–34.6)	−0.2 (−5.5–4.4)	0.043^a^^*∗*^
Fasting insulin (mU/L), mean (SD)	18.7 (6.4–60.2)	18.9 (8.6–74.8)	0.0 (-30.0–14.8)	18.7 (6.4–60.2)	20.0 (9.3–79.6)	0.2 (−32.5–54.3)	0.363^b^
2-hour postprandial insulin (mU/L), mean (SD)	96.4 (26.7)	89.20 (31.84)	−7.27 (25.87)	96.47 (26.70)	90.11 (32.37)	−6.36 (28.14)	0.869^a^
HOMA-IR, mean (SD)	4.8 (1.9–14.2)	5.1 (2.0–19.4)	−0.1 (−10.2–6.2)	4.8 (1.9–14.2)	4.6 (2.2–20.4)	-0.2 (-10.6–14.2)	0.523^b^
FBG (mg/dL), mean (SD)	106.2 (11.3)	102.2 (12.2)	−4.0 (15.4)	106.2 (11.3)	101.1 (12.2)	−5.1 (14.7)	0.578^a^
2-hour postprandial blood glucose (mg/dL), mean (SD)	140.5 (33.6)	146.9 (40.0)	6.5 (35.5)	140.5 (33.6)	140.5 (38.9)	0.1 (42.3)	0.336^a^
DPP-4 (ng/mL), mean (SD)	561.8 (175.8)	598.6 (207.4)	36.8 (113.1)	561.8(175.8)	610.6 (208.5)	48.8 (98.7)	0.515^a^
GA (%), mean (SD)	12.7 (1.7)	12.6 (1.8)	−0.1 (0.8)	12.7 (1.7)	12.6 (1.7)	−0.1 (1.0)	0.937^a^

Notes: comparative analysis using ^a^paired t-test and ^b^Wilcoxon test; ^*∗*^statistically significant if *p* < 0.05.

## Data Availability

The datasets used and/or analysed during the current study are available from the corresponding author on reasonable request.

## Abbreviations

DPP-4: Dipeptidyl peptidase-4
GIP: Glucose-dependent insulinotropic peptide
GLP-1: Glucagon-like peptide 1
GLP-1R: Glucagon-like peptide 1 receptor
HOMA-IR: Homeostasis model assessment of insulin resistance
OGTT: Oral glucose tolerance test
RCT: Randomized controlled trial
T2DM: Type 2 diabetes mellitus.
